# Microwave Imaging for Early Breast Cancer Detection: Current State, Challenges, and Future Directions

**DOI:** 10.3390/jimaging8050123

**Published:** 2022-04-23

**Authors:** Nour AlSawaftah, Salma El-Abed, Salam Dhou, Amer Zakaria

**Affiliations:** 1 Biomedical Engineering Graduate Program, American University of Sharjah, Sharjah P.O. Box 26666, United Arab Emirates; g00051790@alumni.aus.edu (N.A.); selabed@alumni.aus.edu (S.E.-A.); 2 Department of Computer Science and Engineering, American University of Sharjah, Sharjah P.O. Box 26666, United Arab Emirates; 3 Department of Electrical Engineering, American University of Sharjah, Sharjah P.O. Box 26666, United Arab Emirates; aszakaria@aus.edu

**Keywords:** breast cancer, breast tissues electrical properties, microwave breast imaging, passive radiometry, microwave tomography, radar-based imaging

## Abstract

Breast cancer is the most commonly diagnosed cancer type and is the leading cause of cancer-related death among females worldwide. Breast screening and early detection are currently the most successful approaches for the management and treatment of this disease. Several imaging modalities are currently utilized for detecting breast cancer, of which microwave imaging (MWI) is gaining quite a lot of attention as a promising diagnostic tool for early breast cancer detection. MWI is a noninvasive, relatively inexpensive, fast, convenient, and safe screening tool. The purpose of this paper is to provide an up-to-date survey of the principles, developments, and current research status of MWI for breast cancer detection. This paper is structured into two sections; the first is an overview of current MWI techniques used for detecting breast cancer, followed by an explanation of the working principle behind MWI and its various types, namely, microwave tomography and radar-based imaging. In the second section, a review of the initial experiments along with more recent studies on the use of MWI for breast cancer detection is presented. Furthermore, the paper summarizes the challenges facing MWI as a breast cancer detection tool and provides future research directions. On the whole, MWI has proven its potential as a screening tool for breast cancer detection, both as a standalone or complementary technique. However, there are a few challenges that need to be addressed to unlock the full potential of this imaging modality and translate it to clinical settings.

## 1. Introduction

Cancer is a complex disease whose rate of incidence and mortality are rapidly growing worldwide. According to the American cancer society (ACS), around 1.9 million new cases and 600,000 deaths are expected to occur in the United States alone, making cancer the second ranking cause of death globally [1]. For both genders, statistics showed that lung cancer is the most commonly diagnosed cancer type (11.4% of the total cases) and is the leading cause of cancer-related deaths (18.4% of total deaths). Among females, breast cancer is the most commonly diagnosed cancer type and is the leading cause of cancer-related deaths (11.7% of total cancer cases), followed by colorectal and lung cancers. Regardless of the cancer type, early detection is considered crucial in the cancer treatment process as many types of cancer have a high chance of being cured if detected early and treated adequately. For instance, if breast cancer is detected early, the five-year survival rate can become as high as 90% [2]. In this work, we focus on the early detection of breast cancer that has the potential to enhance survival rates, improve the quality of life, and reduce the complications and costs associated with cancer treatment.

Cancer is defined as the rapid growth and proliferation of abnormal cells; these cells undergo changes that disturb the systematic life course of the cell. Such changes arise mainly due to alterations in the genetic programs controlling cell proliferation, relations with neighboring cells, and detection by the immune system. There are various tests that can be performed to detect cell mutations. The simplest of tests can detect only one type of mutation in one gene, whereas more complex tests can simultaneously detect all the types of gene alterations (e.g., substitutions, duplications, insertions, deletions, gene copy number variations, and structural variants, including inversions and translocations). Examples of these tests include allele-specific polymerase chain reaction (PCR), Sanger dideoxy sequencing, pyrosequencing, multiplex ligation-dependent probe amplification (MLPA), mass spectrometry (MS), and fluorescence in situ hybridization (FISH) [3].

Currently, there are several established techniques for breast cancer screening and detection. The most common techniques include clinical breast examination, X-ray mammography, ultrasonography, magnetic resonance imaging (MRI), and positron emission tomography (PET). X-ray mammography is commonly used for early breast cancer diagnosis; however, this modality has several shortcomings. It requires breast compression which can cause the patient pain or discomfort. It is also difficult to distinguish tumors in dense breast mammograms, because both dense breast tissue and tumors appear white in mammogram images. X-ray mammography can also lead to unnecessary, and often invasive, follow-up tests (e.g., biopsies) associated with false positive test results. With regard to radiation exposure, mammography is associated with a small amount of radiation, but the radiation risk is considered irrelevant compared to the advantage of early detection. Breast ultrasonography or sonomammography is a painless technique; however, ultrasound (US) images tend to have low resolution and do not enable one to distinguish between benign and malignant tumors. In addition, sonomammography is mostly used as a secondary technique, usually after a mammography result shows a suspected mass. MRI is typically used for further evaluation of questionable findings. Moreover, MRI is considered the best technique for post-chemotherapy imaging and has the added advantage of being sensitive to imaging silicone breast implants. MRI has better resolution and less operator dependence than US. In addition, it does not use radiation, making it safer than modalities that do, thus allowing use with pregnant patients. On the other hand, MRI is rather costly, making it economically unsuitable as an early screening and detection method. The main advantage of PET is that it can diagnose cancer in the very early stages and scan the entire body for recurrence. However, it tends to have low resolution [4,5,6]. The majority of existing imaging modalities depend on the interactions of electromagnetic waves or acoustic waves with body tissues and fluids. With respect to electromagnetic waves, the majority of the spectrum has already been explored, from the high-frequency end (as in X-rays and PET) to low-megahertz bands (where MRI operates). The portion of the spectrum that is least investigated and has been generating a lot of interest for medical imaging in the past twenty years is the microwave frequency band, which occurs between 300 MHz and 300 GHz [7]. At microwave frequencies, the interaction of electromagnetic signals with matter depends on the material’s dielectric properties, which are the electric permittivity and conductivity. For body tissues, the dielectric properties are related directly to the water content of different biological constituents [8,9,10]. Low-water-content tissues include fat and bones, whereas high water content tissues include muscles, brain, blood, internal organs, and tumors.

Utilizing variations in tissues’ electrical properties, microwave imaging (MWI) has emerged as a technique to produce dielectric maps for various parts of the body. A typical MWI system consists of an antenna array, a microwave signal transmitter and receiver, and a radio-frequency switch to alternate between the different elements of the array [11]. The array surrounds a human organ, which is immersed in a matching medium. During measurements, each element in the array transmits an electromagnetic signal, with the other antennas receiving the reflected waves. The collected measurements are input into an optimization algorithm to produce an image of the dielectric properties of the organ. Some of the biomedical applications of MWI being investigated include brain stroke detection [12], extremities imaging [13,14], and lung cancer detection [15].

This paper focuses on the application of MWI in breast cancer detection. In comparison to normal cells, the water content in tumors is high. This can be attributed to the nature of tumor cells that causes them to retain more fluid than normal cells. This extra fluid, which is in the form of bound water, changes the dielectric properties of breast tissues. In MWI, tumors are detected using scattered or reflected waves, which arise from the differences in dielectric properties between normal and malignant breast tissues [5,16,17,18].

The objective of this paper is to provide a comprehensive up-to-date survey of the developments and current status of MWI as an early breast cancer screening tool along with an overview of the state of the art and future directions. Other recent surveys have been published in the literature. Moloney et al. [19] provided a survey on several imaging modalities used for breast cancer detection, including the potential role of MWI. Misilmani et al. [20] provided a survey focusing specifically on the antenna designs of the MWI systems used for breast cancer detection. Aldhaeebi et al. [21] presented a comprehensive survey of MWI systems used for breast cancer detection focusing on two types of MWI, namely, microwave tomography (MWT) and radar-based techniques. Compared to other surveys, our paper focuses on highlighting the specifications and findings for initial experiments and recent advances in measuring the dielectric properties of breast tissues as well as previous and ongoing developments of MWI systems. In addition, this paper discusses research trends and provides plots demonstrating the number of citations for research publications related to the use of MWI in breast cancer screening among different disciplines. In addition, the paper discusses challenges facing MWI as a breast cancer detection tool and provides an overview of the state of the art and future research directions such as the development of the hybrid and portable MWI systems and using machine learning and deep learning techniques in breast cancer detection.

The rest of the paper is organized as follows. In Section 2, a literature review about the research conducted investigating the dielectric properties of breast tissues and tumors is summarized. Next, in Section 3, current MWI systems for breast cancer detection are categorized and presented. Image reconstruction and MW image quality are discussed in Section 4. The challenges facing MWI as a breast cancer detection tool along with future research directions are outlined in Section 5.

## 2. Dielectric Properties of Breast Tissues

As discussed earlier, the objective of an MWI breast cancer detection system is to use dielectric properties to distinguish healthy breast tissues from cancerous ones. Therefore, understanding the variations of these properties with respect to tissue type and frequency is essential to enable researchers to (i) distinguish between the different types of breast tissues in MWI, and (ii) build experimental phantoms and/or simulation models for testing MWI systems. For this reason, measuring the dielectric properties of normal and cancerous breast tissues have long been the subject of various studies. These measurements are performed mainly ex vivo by extracting a sample of the breast tissue and measuring its dielectric properties using an electrical probe. In this section, a description of the most important studies is presented and summarized in chronological order.

One of the earliest studies to investigate the contrast in dielectric properties of normal and malignant breast tissue in the microwave frequency range was conducted in 1984 by Chaudhary et al. [22]. In this study, the dielectric properties of ex vivo normal and malignant breast tissue were measured at frequencies between 3 MHz and 3 GHz using an RX-meter and a time domain spectrography (TDS) probe. The average of the measurements showed a three to five times increase in the electrical properties of malignant tissues in comparison to those of normal tissues.

In 1988, Surowiec et al. [23] conducted a study to investigate the differences in the dielectric properties of breast cancer tissue. Dielectric measurements were obtained for tissue samples from the central part of the tumor, and surrounding tissue (both immediate and at a distance of 2 cm from the center). The properties were measured using coaxial capacitive sensors at frequencies ranging between 20 kHz to 100 MHz. The samples taken from, and closer to, the center showed increasing values for the dielectric properties.

In 1992, Campbell and Land [24] measured the complex permittivity of female breast tissues at 3.2 GHz using the resonant cavity perturbation method. The authors of this study inspected four tissue types: fatty, normal, benign, and malignant breast tissues. Similar to [22,23], Campbell and Land noted a significant disparity in dielectric values between normal and cancerous tissues; however, their findings showed similarities in the dielectric properties of benign and malignant tumor tissues, hence, they suggested that it might not be possible to distinguish between the two types of tumors based on dielectric properties alone.

In 1994, Joines et al. [25] measured the permittivity and conductivity of healthy and malignant breast tissues at frequencies ranging from 50 to 900 MHz, and their findings were consistent with the aforementioned studies. Furthermore, across the range in which the tissues were examined, the authors observed an average difference in permittivity and conductivity of 233% and 577%, respectively, between healthy and malignant tissues. For these measurements, the authors utilized an open-ended coaxial probe.

These early studies had some limitations in estimating the differences between the dielectric properties of normal and malignant breast tissues, mainly because the sample size was small, only a small range of frequencies was used, there were uncertainties in measurement methods, the differences in dielectric properties between normal, benign, and malignant breast tissues were not studied in detail, and there was inaccurate estimation of the in vivo contrast [21].

Another relevant study was conducted in 2004 by Choi et al. [26]. The authors of this study measured the dielectric properties of normal and metastasized lymph nodes in the breast region. Similar to the study conducted by Joines [25], measurements were performed using an open-ended coaxial probe. Again, their findings conformed with the previous studies and a significant difference in dielectric properties’ values was found between healthy and cancerous tissue.

In 2007, Lazebnik et al. carried out what is arguably the most comprehensive investigation into the dielectric properties of breast tissues [27,28]. This work was a collaboration between the University of Wisconsin—Madison in the United States and the University of Calgary in Canada. What makes this study different from previous examinations of the dielectric properties of breast cancer tissue is that the samples were categorized histologically (i.e., the adipose, glandular, and fibrous connective tissue of each sample was quantified); in addition, Lazebnik and colleagues measured the dielectric properties of three types of breast tissue, namely, normal, benign, and malignant [27,28]. The first study [27] involved 93 patients, while the second study [28] contained samples from 196 patients. The collected samples were then divided into three groups, depending on the percentage of adipose tissue within the sample [5,18]:

- Group 1 contained samples with 0–30% adipose tissue (99 samples);
- Group 2 contained samples with 31–84% adipose tissue (84 samples);
- Group 3 contained samples with 85–100% adipose tissue (171 samples).

The findings of Lazebnik et al. conformed with those of Campbell and Land [24], i.e., there is significant heterogeneity in the dielectric properties of breast tissue (refer to Table 1). This was attributed to the composition of the tissue samples, as most breast tissue samples in previous studies were taken from surrounding glandular tissue which has a higher adipose content than tumor glandular tissue. Therefore, the dielectric heterogeneity of breast tissue was underestimated in previous studies.Accordingly, Lazebnik et al. concluded that the dielectric properties of breast tissue depend on the location from which the sample was taken and adipose content. In Lazebnik et al.’s [28] second study, a larger sample size and a wider frequency band (0.5–20 GHz) compared to previous studies were investigated to improve accuracy and ensure that the findings were more reflective of the population. The measured dielectric values for malignant tissue agreed well with previous studies by Chaudhary [22], Surowiec [23], and Joines [25]. Without adjusting for adipose or fibrous connective tissue, Lazebnik et al. observed a 10:1 contrast between normal high adipose tissue and malignant tissues [5,18,27,28]. In addition to the ex vivo studies, Meaney et al. estimated the dielectric properties of cancer-free breast tissue in vivo using a tomographic microwave imaging system in 2000. The results of this study showed that the average permittivity values at 900 MHz were significantly higher than those reported by Joines et al. [25]. A detailed chronological summary of the studies discussing dielectric properties of breast tissues is provided in Table 2.

## 3. Microwave Breast Imaging

As discussed in Section 2, the use of microwave imaging to detect breast cancer is achievable due to the difference in the electric properties between healthy and cancerous tissues. Although the penetration depth in living tissues is considered limited in the microwave frequency range, in MWI, the electromagnetic signals need to propagate only for a few centimeters in breast tissues to provide the information needed for diagnosis [17,33].

The use of microwave imaging offers the following several advantages over other imaging modalities [34]:

- The use of low-power, non-ionizing electromagnetic radiation, which does not pose a health risk for patients.
- Hardware utilized is relatively inexpensive.
- The system can be used to detect breast cancer in men.
- The measured signals are sensitive to all tumors and offer a specific contrast for malignancy.
- Using MWI, breast cancer can be detected at an early stage.
- The measurement procedure involves minimal discomfort for patients.

**Figure 1:**
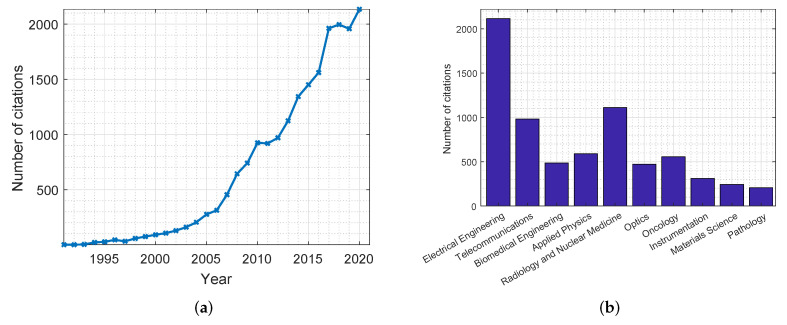
Citations of publications on the use of MWI in breast cancer screening applications from the Web of Science databases. Citations are presented: (**a**) per year; (**b**) per discipline.

Due to the aforementioned advantages, the use of MWI has been gathering quite a great deal of attention over the past years, leading to a significant amount of research in the field. Figure 1 shows plots highlighting the number of citations for publications related to the use of MWI in breast cancer screening applications. These statistics were retrieved from the Web of Science databases. As can be noticed in Figure 1a, there has been an increasing interest in this topic in recent years. Furthermore, Figure 1b shows a distribution of the disciplines in which MWI is most mentioned, with the field of electrical engineering holding the greatest number of cited publications. Table 3 and Table 4 summarize the most significant research conducted in the development of microwave systems for breast cancer detection. Table 3 includes simulation and phantom studies, while Table 4 includes a summary of clinical trials.

**Figure 2:**
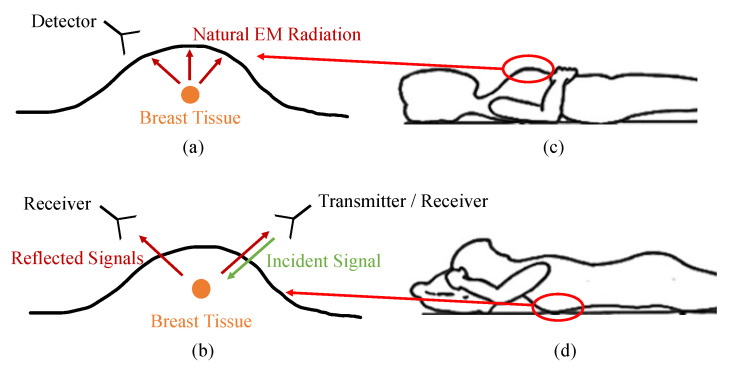
Methods of microwave breast imaging. The figures on the left show (**a**) passive versus (**b**) active approaches. The figures on the right show patient’s orientations for (**c**) planar systems (supine position) versus (**d**) cylindrical systems (prone position).

MWI for breast cancer detection can be classified into two groups: passive and active. The main difference between both approaches is as follows; in passive systems, the natural electromagnetic (EM) radiation emitted by living tissues is measured. On the other hand, in active systems, an electromagnetic signal is transmitted from a source into the tissues, and the reflected signals are measured. These two approaches are depicted in Figure 2a,b. In the following subsections, details regarding these MWI systems are explained and discussed.

### 3.1. Passive Microwave Imaging

Passive MWI relies on the difference in temperature between healthy and cancerous tissues, as tumors tend to have higher temperatures due to increased vascularization [33]. Microwave radiography, also known as thermometry or thermography, incorporates radiometers to measure temperature differences in the breast. Radiometry for breast imaging has been investigated for many years and tested clinically. A good example of such systems is ONCOSCAN, which was used by Carr et al. [35] in accompaniment with mammography to test the thermal activities of breast tissues. The detection of temperature differences using ONCOSCAN was utilized as a substitute for breast biopsies for diagnosis.The readings obtained from the 130 subjects that were scanned indicated that the system had high predictive power when coupled with mammography. In 2004, Stec et al. [36] developed a system to measure and model deep-seated temperature distributions in biological tissues by means of multi-frequency microwave thermography. The mapping of temperature variations as a function of depth obtained from this model was consistent with the findings of known physical models.

### 3.2. Active Microwave Imaging

Active approaches of MWI involve radiating the breast with electromagnetic signals at microwave frequencies, detecting the energy reflected or scattered due to the breast, then processing the collected data to form images. For breast cancer detection, active MWI can be divided into two types: tomography and radar-based imaging [5].

#### 3.2.1. Microwave Tomography

Microwave tomography (MWT) can be best defined as a method that produces two-dimensional (2D) slices (tomo) or images of the dielectric properties of an object of interest (OI) by measuring the electromagnetic field perturbations around this OI. MWT systems typically consist of an imaging chamber in which an array of antennas is placed surrounding the OI. The imaging chamber is filled typically with a matching medium to couple most of the microwave electromagnetic energy with the breast, which improves the system’s performance. During measurements, each antenna transmits a continuous wave (CW) single- or multi-frequency electromagnetic signal. Due to the differences between the dielectric properties of the OI and the matching medium along with property variations inside the OI, the electromagnetic fields are scattered and measured by the other non-transmitting antennas. The measurements are used as input for specialized algorithms to create the 2D images of the dielectric properties.

**Figure 3:**
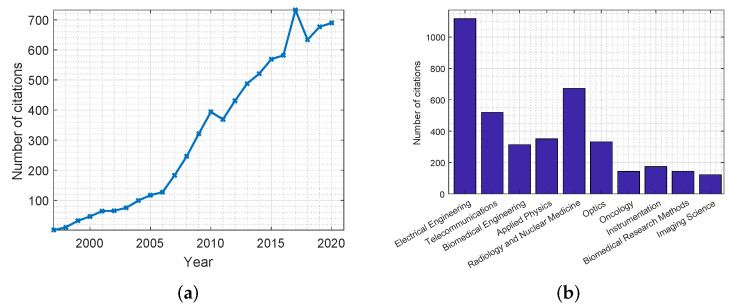
Citations of publications on the use of MWT in breast cancer screening applications from the Web of Science databases. Citations are presented: (**a**) per year; (**b**) per discipline.

The use of MWT dates back to the 1970s with some of the earliest reports of biomedical applications being in the early 1980s [18]. Several research groups investigated MWT for breast cancer detection. Figure 3 shows plots that highlight the number of citations for publications related to MWT in breast cancer screening applications. These statistics were obtained from the Web of Science databases. As can be concluded, for the past three decades there have been many publications in this field, with many of them in journals related to the discipline of electrical engineering.

**Figure 4:**
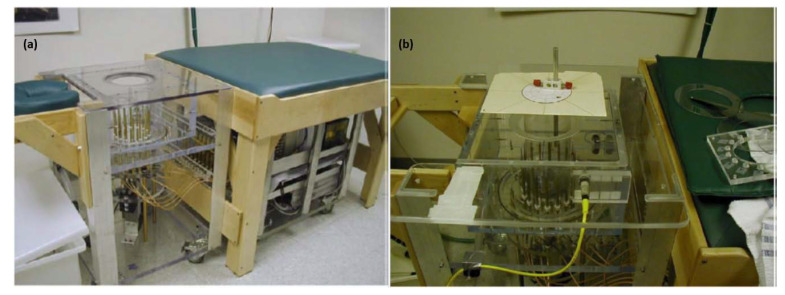
(**a**) MWT multi-frequency prototype; (**b**) typical phantom experiment with liquid containers suspended from above the tank and integrated with an alignment fixture for accurate positioning. Reprinted with permission from [38].

The first clinically tested MWI system was reported by Meaney’s group at Dartmouth College (the setup used is shown in Figure 4) [29,37,38,39]. The group conducted several variations of the study, each time improving upon the previous iteration. The prototype MWI system consisted of a 16-element transceiver monopole antenna array operating at frequencies between 300–1000 MHz. The participants’ breasts were imaged with the subject in a prone position on the examination table with the breast pendant in a saline solution. Each imaging session lasted about 15 min per breast and included full tomographic data acquisition from seven different array positions (beginning at the chest wall and moving anteriorly toward the nipple) at seven different frequencies per array position. The preliminary findings of this study revealed that a relationship existed between breast permittivity and radiological breast density.

In another iteration of their experiment, Meaney et al. [37,38,39] used their MWT system but changed the matching medium from a saline solution to a glycerin/water mixture in an attempt to optimize the coupling liquid, as this mixture was found to be better matched to the electrical impedance of the breast. This change helped in reducing coupling noise between the array elements, and thus providing more accurate images than the group’s earlier study. In the first part of the new experiment, the study involved imaging a set of cylindrical phantoms with two tumor-like inclusions (1 cm and 2 cm in diameter, respectively) positioned in the imaging array. The phantom studies were intended to test the accuracy with which the developed system could estimate the distribution of dielectric properties. The second part of the study involved evaluating the clinical feasibility of the developed system and assessing the microwave properties of the normal breast in vivo in 43 female subjects.The overall results of this study were promising, particularly the clinical trials, as the system was able to detect tumors as small as 1 cm in diameter, confirming the potential of MWI in early detection of breast cancer [37,40]. More recently, Meaney et al. [16,38,39] applied their MWI imaging system to monitor the treatment progress of women undergoing neoadjuvant chemotherapy (NCT) for locally advanced breast cancer. Eight patients were imaged throughout the course of their treatment. Microwave property values were extracted from the regions of interest (ROIs) followed by statistical analyses to assess short-term (30 days) and longer term (four to six months) dielectric property changes. The results demonstrated changes in microwave properties that agreed well with the overall NCT treatment response.

Another development in breast cancer MWT involves using magnetic nanoparticles (MNPs) as contrast agents to improve imaging accuracy and specificity [40]. Bucci et al. [41] addressed the problem of optimizing the design of such an MWI system. The authors used their developed system to image two breast phantoms with tumor-like inclusions obtained from the repository of the University of Wisconsin [27,28]. Their analysis reported that by using 50 antennas on each of the eight measurement circles surrounding the breast, it was possible to measure the differential fields arising from the MNPs at the tumor location inside the breast. Thus, an MNPs-enhanced MWI system would be able to detect cancerous tumors.

#### 3.2.2. Radar-Based Microwave Imaging

In contrast to MWT, which measures signals due to the changes in the whole matter of the OI, radar-based MWI constructs an image using only reflected signals due to sudden variations in the electrical properties in the OI [5]. In breast cancer detection, radar-based MWI uses the reflected waves that arise due to the differences in the dielectric properties between normal and malignant breast tissues to identify the presence and location of tumors [42]. Radar-based approaches use ultra-wideband (UWB) signals to satisfy the resolution requirement while maintaining adequate signal penetration as tissue conductivity increases with frequency [33]. It has been found that the frequency range between 1 and 10 GHz is the optimum band for this application [43]. In a UWB radar configuration, transmitting antennas radiate short-duration bursts of microwave energy into an OI. If a dielectric discontinuity is encountered by the traveling wave, it is reflected. The scattered signals are recorded by the receiving antennas and are used to estimate the existence, location, and characteristics of the structure that is causing the dielectric discontinuity [42].

In comparison to MWT systems, UWB imaging systems have several advantages, including [33,43]:

- Computationally less expensive: MWT has computational challenges as it involves solving an inverse scattering problem to reconstruct an image for the complete profile of the breast’s dielectric properties. However, radar-based MWI does not have such computational challenges because the objective is to simply detect the presence and the location of the backscattered energy source, such as tumors, which occurs due to the difference in dielectric properties between normal and cancerous tissues.
- Higher resolution: Using radar-based imaging systems, a precision of less than 5 mm is expected which is good enough for early detection and localization of breast cancer. This is due to the use of UWB signals. In MWT systems, CW single- or multi-frequency signals are utilized, which limits the resolution.
- Better specificity: Radar-based MWI is able to detect if the lesion is malignant or benign. The scattered waves from benign tumors are not as strong as the ones recorded from malignant tissues. The reason behind this is that the dielectric properties of benign tumors are similar to normal tissues, but different than those of malignant tumors.

**Figure 5:**
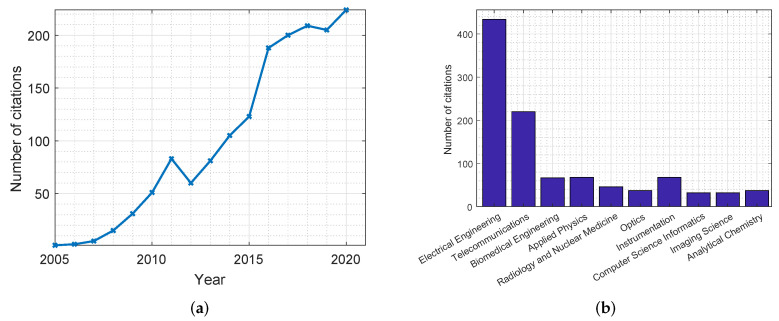
Citations of publications on the use of radar-based MWI in breast cancer screening applications from the Web of Science databases. Citations are presented: (**a**) per year; (**b**) per discipline.

In Figure 5, two plots are produced that show the number of citations for publications related to radar-based MWI. Similar to the previous plots, these statistics were collected from the Web of Science databases.

**Figure 6:**
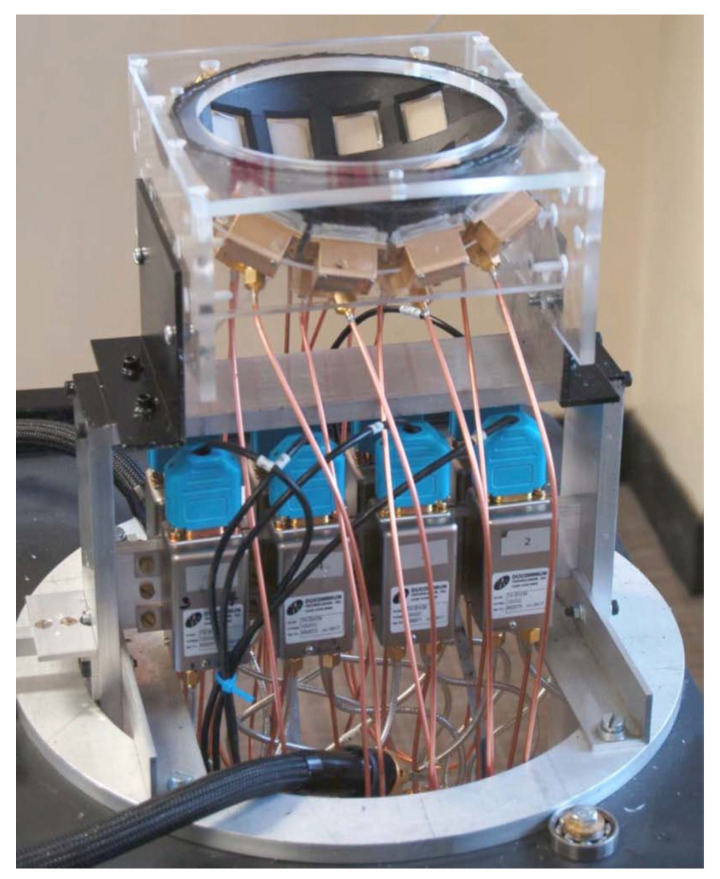
UWB radar for breast cancer detection setup developed by the University of Bristol team. Reprinted with permission from [46].

Developed radar-based MWI systems can be classified into five groups [40]:

- Confocal microwave imaging (CMI): This system was first introduced in 1998 by Hagness et al. [44]. It relies on pulsed confocal techniques and time-gating to enhance the detection of tumors while suppressing tissue heterogeneity and absorption effects. CMI involves using an array of antennas to focus a UWB signal at a potential tumor site, then using the same antennas to collect the scattered microwave energy from the tumor by refocusing it at the point of transmission origin [44]. The work of Hagness et al. [44] involved a finite-difference time-domain (FDTD) solver to simulate the dielectric properties of normal and malignant breast tissues (using published dielectric properties values). The FDTD simulations showed that tumors as small as 2 mm in diameter could be detected [44]. In 1999, Hagness and Taflove [45] improved the previous design by using a resistivity bowtie antenna, and performing 3D-FDTD simulations [33]. Further numerical and experimental research in using the CMI system was carried out by Hagness’s group at the University of Wisconsin—Madison.
- Multi-static adaptive (MSA) system: Another series of high-impact radar-based MWI studies were performed by a research group in the University of Bristol [46,47,48]. The developed system used a real aperture array of UWB antennas (the developed system is shown in Figure 6). In 2009, the Bristol University group presented a UWB MWI system for the detection of breast cancer that consisted of 16 UWB aperture-coupled stacked-patch antennas located on a section of a hemisphere. The antennas were arranged this way to improve their conformation to the curve of the breast. The system was tested using realistic 3D breast phantoms and was successful in detecting tumors of 4 to 6 mm in diameter [47]. In 2010, the group conducted a large initial trial of its 31-element prototype radar system at the Breast Care Center in Bristol, UK. Although this system yielded excellent results, the outcomes of the clinical trial were mixed. Some successful detections were made as judged by an independent clinician but the repeatability of these results was lacking. The irreproducibility of the results was attributed to slight patient movements during the 90-second scans together with some uncertainties introduced by variations in blood flow and temperature. To overcome these inadequacies, the group developed a 60-antenna array system, where the increased antenna density was meant to improve the system’s immunity to clutter and shorten acquisition time to 10 seconds. This system underwent extensive clinical trials at the Breast Care Center at Frenchay Hospital, Bristol, UK. The rapid data acquisition improved the accuracy of the obtained images while also providing a clinical experience that is more convenient and acceptable to patients.
- Tissue-sensing adaptive radar (TSAR): This system was developed and tested by Fear’s group at the University of Calgary et al. [49,50]. TSAR requires two scans of each breast. The breast is suspended through a hole in the examination table into a tank that contains the antenna and is filled with a coupling fluid. The first scan determines the overall location of the breast volume relative to the tank, utilizing the first reflection received at the antenna. The second scan is performed in a coronal fashion progressing from nipple to chest wall providing the data for the tumor detection algorithm [50]. Clinical results showed that TSAR has an ability to detect and localize tumors with sizes greater than 4 mm in diameter. However, this system faced some challenges such as the large reflections caused by the skin, the development of appropriate antennas, and the requirement to develop high-speed electronics for real-time imaging. Current work on TSAR includes development of appropriate sensors, exploration of practical implementation issues, improvement of imaging algorithms, and testing on breast models [40,42].
- Microwave imaging via space–time (MIST) beamforming: This methods involves the sequential transmission of UWB signals from antenna placed near the breast surface. The received backscattered signals are spatially focused using a space–time beamformer. Due to the significant contrast in the dielectric properties of the normal and malignant tissue, localized regions of large backscattered energy levels appear in the reconstructed images, which correspond to malignant tumors [51]. The first MIST system was introduced by Hagness et al. at the University of Wisconsin—Madison [52]. This system yielded promising results in the detection and localization of very small synthetic tumors embedded in breast phantoms. In addition, Bond et al. [51] developed an MIST system for the detection of millimeter-sized tumors in the breast tissue. This system was made of a planar array of 16 horn antennas that transmitted UWB microwave signals consecutively; this design improvement enhanced the robustness against measurement variations, which resulted in clearer imaging of tumors. Further improvements to the system enabled the system to localize, identify, and resolve multiple tumors [42,51].
- Holographic microwave imaging (HMI): In this approach, microwave imaging is performed on two stages: recording of a sampled intensity pattern followed by image reconstruction [53]. The recording of a sampled intensity pattern is performed by combining the signal scattered by the object, such as a tumor, and a reference signal. At microwave frequencies, the reference signal is electronically synthesized [54]. Smith et al. [53,54,55] proposed several HMI systems. Compared to other radar-based MWI techniques, HMI has the ability to produce real-time images at significantly lower cost because it does not require expensive ultra-high-speed electronics. Experimental results using breast phantoms consisting of skin, fat, and embedded tumor-like inclusions revealed the potential of this approach. However, further validation is still required before this technique can be translated to clinical settings. Wang et al. [56,57] provided significant contributions to the investigations into HMI systems. They proposed a 2D holographic MWI array (HMIA) system for early breast tumor detection. The first system they proposed was designed for operations at a single frequency (12.6 GHz) and included one transmitter and an array of 15 receivers placed under the breast phantom. The breast phantom used for testing consisted of homogeneous normal breast tissue, a small embedded malignant tumor, and the skin. The advantage of this system is that it does not require a matching solution medium; thus, air was used between the antennas and breast phantom. The experimental results showed that small tumors with diameter less than 5 mm at different locations could be successfully detected by using the proposed 2D HMIA technique [56]. Their second experiment aimed at investigating the feasibility and effectiveness of combining compressive sensing (CS) with holographic MWI (CS-HMIA). Their findings revealed that CS-HMIA is capable of detecting randomly distributed inclusions, of various shapes and sizes, using smaller number of sensors and shorter scan times [57]. In a more recent study, Wang [58] developed a multi-frequency HMI system and investigated the feasibility and effectiveness of the proposed algorithm for breast imaging. The effectiveness and accuracy of the multi-frequency system was tested and compared to a single-frequency HMI system. The comparative study showed that the multi-frequency HMI could identify and reconstruct small tumors accurately, even when embedded in dense tissue. All of these findings showed that the multi-frequency HMI system has potential as a microwave diagnostic technique.

**Table 3:** Summary of relevant phantom and simulation microwave imaging studies for breast cancer detection.

Author (Year, Location)	MWI Method	Study Type & Dimensions	Frequency Range	Measurement System	Findings
Dobrowolski et al. [36] (2004 Military University of Technology	Passive, radiometry	Numerical simulation and phantom study: normal tissues (beef meat) 2D	1.5–4.4 GHz	Three-band radiometer with mini hypodermic probes with platinum RTD elements. The modeling was performed by numerically solving the thermal radiation transmission equation as a function of brightness.	- As the brightness temperatures fluctuate randomly due to the nature of thermal radiation, the deep-seated profile of temperature distribution estimated from them also fluctuated randomly. - The numerical error was determined using a Monte Carlo technique and the obtained results indicate a possibility of noninvasive detecting and measuring of spatial temperature distribution inside a human body by means of multi-frequency microwave thermograph.
Bardati and Iudicello [59] (2007, University of Rome Tor Vergata)	Passive, radiometry	Numerical simulation 3D	-	Simulation performed using standard Penne equation and steady state bio-heat equation.	- Radiometer output (difference signal or over temperature) was shown to be a function of tumor depth and size. - Radiometric visibility was found to decrease with tumor depth. - A 10 mm lesion is radiometrically visible if it is no deeper than 2 cm.
Zhurbenko et al. [60] (2010, Technical University of Denmark)	Passive, radiometry	Phantom study 3D	300 MHz–3 GHz	Thirty-two monopole-type antenna array system.	- The dielectric properties for water filled spheres were estimated and presented in 3D colormaps. - A reasonable estimation for the locations and shapes of objects-of-interest was obtained.
Hagness et al. [44] (1998, University of Wisconsin)	Active, CMI	Numerical simulation 2D	-	Monopole antenna and published dielectric properties values were used in 2D FDTD computational electromagnetics analysis.	- In the simulation study, malignant tumors as small as 2 mm in diameter can be detected in the presence of the background clutter generated by tissue heterogeneity.
Hagness and Taflove [45] (1999, University of Wisconsin)	Active, CMI	Numerical simulation 3D	-	Wide-band bowtie antenna and published dielectric properties values were used in 3D FDTD analysis.	- Simulations proved that the system was found capable to detect early-stage malignant breast tumors. - Malignant tumors are typically asymmetrical while most benign masses are well-circumscribed and compact. - The 3D FDTD simulations indicated the possibility of distinguishing between benign and malignant tumors based on the characteristics of their microwave backscatter response.
Bulyshev et al. [61,62] (2000–2001, Carolinas Medical Center)	Active, MWT	Numerical simulation 2D, 3D	2 GHz, 3.5 GHz, and 5 GHz	The Helmholtz equation was used for solving direct problems and the gradient method was used for inverse problem solving.	- Imaged regions close to the array structures, including the malignant zone and skin, were clearly visible. - Tumors of size ≥ 1 cm were clearly detected. - Structures located deeper than 3–4 cm beneath the surface neither appeared on the image nor affected the imaging of the upper layers. - The optimal mesh should be twice as wide as the array. - A smaller mesh tended to distort images. - 3D images of breast can be obtained on the tomographical system in low GHz region with quality sufficient for the small size tumor detection.
Stuchly et al. [63,64] (2001–2002, University of Victoria)	Active, CMI	Numerical simulation 2D, 3D	-	In the planar system configuration, the patient is oriented in a supine position and a resistively loaded bowtie antenna is used to scan the breast to create a synthetic planar array. In the cylindrical configuration, a resistively loaded dipole antenna was used to scan breast with the patient oriented in a prone position and the breast extending through the examination hole.	- CMI was found to be a feasible tool for detecting and localizing breast tumors in 3D. - Both system configurations showed similar efficiency in detecting and localizing breast tumors.
Hagness et al. [52] (2003, University of Wisconsin)	Active, MIST	Phantom study	1–11 GHz	A planar synthetic array of compact UWB antennas are placed on breast phantom with a small (<0.5 cm) synthetic tumor embedded. A data-adaptive algorithm removes the artifact caused by backscatter from the skin–breast interface. The signals were passed through a 3D space-time beamformer designed to image backscattered energy as a function of location.	- The developed system yielded promising results in the detection and localization of very small synthetic tumors embedded in breast phantoms.
Bond et al. [51,65] (2003–2005, University of Wisconsin)	Active, MIST	Phantom study 2D	-	A planar array of 16 horn sensors and breast phantoms based on anatomically realistic MRI-derived FDTD models of the breast. A data-adaptive algorithm for removing artifacts from backscatter from the skin–breast interface.	- Small lesions can be detected with high sensitivity regardless of location in the breast. - Small tumors embedded in heterogeneous normal breast tissue are successfully detected in a wide range of numerical breast phantoms. - The imaged backscatter from a 2 mm diameter tumor stood out significantly above the clutter generated by the inherent variations in the fibroglandular and adipose composition of the breast.
Xie et al. [66] (2006)	Active, MSA	Numerical simulation 3D	-	An aperture array transmits and receives microwave pulses. A two-stage data adaptive robust Capon (RCB) algorithm was adopted along with a realistic 3D breast model simulated by the FDTD method.	The system showed better resolution and noise rejection capabilities than existing methods.
Smith et al. [53,55,67] (2006–2013, Northumbria University)	Active, HMI	Phantom study 2D	9.4 GHz	Transmitting and receiving antenna were used along with a simulated breast phantom with tumor-like inclusions.	- HMI has the ability to produce real-time images at significantly lower cost because it does not require expensive ultra-high-speed electronics. - Experimental results using simulated breast phantoms provide confidence in the potential of this approach. - More validation is required on the theory and proof-of-concept for medical applications.
Galvin et al. [68] (2010, University of Ireland Galway)	Active, MIST	Phantom study 2D	-	- A planar array of 16 horn sensors and breast phantoms based on anatomically realistic MRI-derived FDTD models of the breast. - Both the artifact removal algorithm and the beamformer from Bond’s system [69] were modified to provide for multi-static data.	- The system successfully detected the presence of small tumors (5 mm in diameter) at various depths within the heterogeneous breast tissue. - The quasi-multistatic system produces a significantly improved signal-to-clutter ratio when compared to the traditional monostatic MIST beamformer.
Son et al. [70,71] (2010–2012, Korea)	Active, MWT	Phantom study 2D, 3D	0.5–3 GHz	16 monopole transmitting, receiving (TRx) antennas in plane circular arrangement with breast pendant in coupling liquid. Two types of phantoms were used, circular and cylindrical.	- The presented 2D MWT system demonstrated good sensitivity and reasonable spatial resolution of the reconstructed images of the breast and tumor phantoms. - The scattered signals from the small spherical tumor were much smaller than in the case of the cylindrical tumor. - The system was able to detect and reconstruct an image of a 5 mm in diameter spherical tumor phantom.
Aguilar et al. [72] (2013, University of Wisconsin- Maddison)	Active, CMI	Numerical simulation and phantom study 3D	1.36–3.03 GHz	32 multi-band miniaturized slot-loaded patch antennas in a planar layout.	- The study elucidated the trade-off between miniaturization via slot-loading and gain. - The study revealed how the gain of the miniaturized patch antennas varies with the substrate dielectric constant and thickness. - The results of the computational study suggested that miniaturized antennas are suitable candidates as array elements for multi-band microwave breast imaging systems where unidirectional radiation, environmental shielding, and dense spatial sampling of scattered fields are desired.
Wang et al. [56,57] (2013–2018, Auckland University of Technology and Hefei University of Technology)	Active, HMI	Numerical simulation and phantom study 2D	12.6 GHz	16-element and 64-element uniform sensor array and breast phantoms based on published dielectric properties with air as the coupling medium. The split Bregman and orthogonal matching pursuit algorithms were applied.	- Small tumors of diameter < 5 mm at different locations could be successfully detected. - Both simulation and experimental results demonstrated that CS-HMAI can produce high-quality images and detect arbitrarily shaped small inclusions with random sizes and locations by using significantly fewer sensors and scanning times than regular HMAI.
Augusto et al. [73] (2016, Pontifical Catholic University of Peru)	Active, HMI	Phantom study 2D	2–15 GHz	Both confocal and holographic system algorithms used single Vivaldi antenna for transmission and reception along with breast phantom with tumor-like inclusions.	- Both the confocal and holographic algorithms demonstrated viability for the detection of tumors of diameter ≥ 15 mm. - In the confocal algorithm, the concern is the contrast, while for the holographic algorithm, the concern is locating the tumor phantom without imaginary targets.
Bucci et al. [41] (2015, University of Naples Federico)	Active, magnetic nanoparticle-enhanced MWI	Numerical simulation and phantom study 2D	2 GHz	Magnetic nanoparticles used as contrast agent along with breast phantoms with tumor-like inclusions.	- The analysis presented provided an optimum design of a measurement device devoted to the implementation of this technique. - Using 50 antennas on each of the eight measurement circles present in the design allowed the measurement of the differential field arising in a MNP-enhanced MWI experiment accurately, which in turn allowed the detection of cancerous tumors.
Meo et al. [74] (2017, University of Pavia, Italy)	mm-wave frequency system	Numerical simulation 2D	26.5–40 GHz	32 antennas in conformal layout and the radiators are open-ended WR28 waveguides. Bio-heat equations and F-DMAS algorithm for image reconstruction.	- A penetration depth of a few cm was achieved. - Conformal layout of antenna better than linear layout. - Estimated tumor position (41 mm) in close agreement with theoretical depth (40 mm).
Hammouch et al. [75] (2019, Mohammed V University in Rabat)	Active, CMI	Numerical simulation 2D	3.1–14 GHz	Microstrip patch antenna.	- Results demonstrated the applicability of using CMI for monostatic UWB radar system in breast cancer detection. - Results showed that CMI algorithm is valid for detection and localization of breast tumors. - The method is of lower cost and is considered non-invasive radiation compared to other screening methods.
Islam et al. [76] (2019, Universiti Kebangsaan Malaysia)	Active, radar-based	Numerical simulation and phantom study: lab-made heterogeneous tumors 2D	2.80–7.00 GHz	A compact side slotted tapered slot UWB antenna is designed in which the slot antenna side is minimized. The antenna array, side-slotted Vivaldi, will be sending microwave pulses directed toward the suspected area. (9 antenna array, 8 × 50 scanned position).	- The proposed UWB antenna-based MWI system provided real-time detection of breast tumors. - A significant variation of backscattered signal exploits tumor cells of a breast. - Tumor cells inside breast are detected using the side-slotted Vivaldi antennas.
Srinivasan et al. [77] (2019, SSM Institute of Engineering and Technology)	Active, dielectric substrate	Numerical simulation 2D, 3D	2.45 GHz	Wearable jeans material used as dielectric substrate in which an antenna is designed as a sandwish model with slot loaded over patch and ground plane made of copper.	- The study proposed a low-cost textile wearable antenna for breast cancer detection.
Soltani et al. [78] (2019, University of Waterloo)	Active, microwave-induced thermoacoustic imaging (MITAI)	Numerical simulation 3D	2.45 GHz	Three different breast tissue types along with a tumor were placed in a tank filled with castor oil. The tissues were irradiated by a 2.45 GHz pulsed microwave source from a rectangular waveguide. The generated heat and pressure gradient in the biological tissue due to the electromagnetic wave irradiation were evaluated.	- Thermoacoustic imaging is used to obtain maximum temperature and pressure variation at tumor. - Location of tumor is related to detecting performance in MIRAI method. Tumors located in fatty tissues tend to be easier to detect than other which are located in transitional tissues. - Size of tumor plays a role in detection and performance of MITAI technique. - MITAI method can detect tumors of 0.5 cm diameters.
Sheeba et al. [79] (2019, Sathyabama Institute of Science and Technology)	Active	Numerical simulation and phantom study: human skin and breast model (normal and malignant tissues) 2D	2.4 GHz	Flexible soft-wear hexagonal patch antenna with jean substrate (with and without slot).	- In simulation, the presence and absence of tumor as 20.3 A/m^2^ and 19 A/m^2^ and gain as 7.20 and 7.25 dB was noted in breast model in CST. - The existence of the tumor is 25.9 A/m^2^ and the nonexistence of the tumor is 21.1 A/m^2^ and the gain is 6.91 dB for with and without tumor is 6.95 dB is noted using CST.
Geetharamani et al. [80] (2019, Anna University)	Active, metamaterial-inspired Terahertz	Numerical simulation and phantom study: normal and malignant tissues 2D	1 THz	Metamaterial-inspired THz antenna of a simple rectangular patch configuration integrated with complementary split ring resonator (CSRR).	The experimental technique proposed was able to detect the tumor in the tested breast tissue model.
Islam et al. [81] (2019, Universiti Kebangsaan Malaysia)	Active	Numerical simulation and phantom study: lab-made realistic heterogeneous tumors 2D, 3D	2.7–11.2 GHz	Index Near-Zero Metasurface Loaded High Gain Antenna, 16 antenna arrays, 64 × 50 scanned position.	- An efficient, viable, and low-cost testing system is proposed to detect multiple abnormalities of tumor clusters inside the breast.
Wang [58] (2019, Hefei University of Technology)	Active, HMI	Numerical simulation 2D, 3D	1–4 GHz	Small waveguide antenna simulated as a transmitter and detector.	- Multi-frequency HMI algorithm can detect small breast lesions with higher accuracy compared to the single-frequency HMI. - Proposed method improves image resolution which aids in developing vision tool for microwave diagnostic techniques.
Felício et al. [82] (2020, Universidade de Lisboa)	Active, radar-based	Numerical simulation and phantom study 2D, 3D	2–5 GHz	Dry setup, fixed cylindrical balanced antipodal Vivaldi antenna (BAVA) configuration with a diameter of approximately 120 mm, artifact removal algorithm developed, webcam used for breast 3D surface reconstruction.	- Obtained very good detection of the tumor in different positions (maximum positioning error was 10.8 mm) - Lower contrast observed in fibroglandular tissue. - Feasible setup for operation in real exams.
Abdollahi et al. [83] (2020, University of Manitoba)	Active	Numerical simulation 2D	0.8, 0.85, 0.9, and 0.95 GHz	Perfect electric conductor (PEC) chamber and 2D transverse magnetic (TM) transceivers in a circular array.	- Tumors were well localized at all frequencies and with all incorporated prior-information maps. - The highest AUC of over 0.99 was obtained for breast model II (fatty breast) while the lowest AUC values correspond to breast model I (heterogeneously dense breast) - For all three breast models, no artifacts were created inside the fatty tissue.
Oloumi et al. [84] (2020, University of Alberta)	Active, circular synthetic aperture radar (CSAR)	Numerical simulation and phantom study 3D	1 MHz	UWB radar system (AVTECH AVP-3SA-C pulse generator, Vivaldi antennas, and sampling oscilloscope), a breast phantom, and a matching liquid container (vegetable oil)	- Results from measurements and comparison with MRI image of the phantom demonstrated the capability of this method to improve the image quality. - This experiment did not consider the effect of skin and adipose tissue, but numerical simulations showed that the distortion of the signal was not significant for the given operating frequency.
Kumari et al. [85] (2020, National Institute of Technology, Delhi, India)	Active, near-field indirect HMI	Phantom study2D	8.5 GHz	Two Vivaldi antennas used as transmitter and receiver, directional coupler, variable attenuator, phase shifter, a magic Tee, power sensor.	- The developed system was able to identify and locate tumors up to the minimum size of 4 mm and maximum depth of 25 mm in the phantom.
Ahmed et al. [86] (2020, Middle Technical University, Baghdad, Iraq)	Active, radar-based	Numerical simulation and phantom study 3D	6.1–12 GHz	18 Peano patch antenna array arranged in a semi-sphere designed by CST Microwave studio simulator.	- More than one antenna was needed around the breast to improve the resolution of the image of the image. - The antenna showed strong pattern of omnidirectional radiation.
Iliopoulos et al. [87] (2020, University of Rennes, France)	mm-wave frequency system	Numerical simulation and phantom study 2D	20–40 GHz	Transmitting and receiving antennas were manufactured in-house using laser ablation	- Good agreement between simulation and theoretical results.
Rahpeima et al. [88] (2020, K. N. Toosi University of Technology, Tehran, Iran)	Active, MITAI	Numerical simulation 3D	2.45 GHz	Simulations were performed using the COMSOL software.	- More temperature increase detected in tumor area than in the other tissues. - Tumor size did not have a significant impact on the efficiency of detection. - Very small tumors with a radius of 0.25 cm were detectable. - Tumors located in fatty tissues were much easier to detect than those in the glandular tissues. - With augmentation of the irradiation power level or increasing the pulse width, stronger acoustic waves are produced to make tumor detection easier.
Miraoui et al. [89] (2020, University Mustapha Stambouli, Mascara, Algeria)	Active, radar-based and ANN	Numerical simulation 2D	4 GHz	Bow-tie antennas for the transmission and reception, CST software used for the simulation.	- The simulation results depicted that the ANN presented more precision in the detection and localization of tumors.
Coşğun et al. [90] (2020, Bolu Abant Izzet Baysal University, Bolu, 14030 Turkey)	SPION-enhanced MWI	Numerical simulation and phantom study 2D	1.9–2.02 GHz	18 vertical dipole antennas placed below the metallic surface of the bed and equidistantly distributed. Breast and antenna suspended in epoxy resin. SPION tracer was used.	- The proposed technique detected much smaller tumors as compared to the operation wavelengths between 1.8 cm and 7.5 cm for the simulation models. - Despite the electric field difference, the factorization method was able to adequately reconstruct spatial variation of SPION tracers in the frontal plane of the breast.
Kaur and Kaur [91] (2020, Thapar Institute of Engineering and Technology, Patiala, India)	Active, synthetic aperture radar (SAR)	Phantom study 2D	4.9–10.9 GHz	Three-layered stacked aperture coupled microstrip antenna (SACMPA) with a defected ground structure, a vector network analyzer (VNA), and an anechoic chamber.	- The specific adsorption rate on the breast phantom at the frequencies of 5.7 GHz was 0.271 W/kg and at 6.5 GHz is 1.115 W/kg for 1 g of body tissue. This proved that the antenna was safe for human exposure (below 1.6 W/kg for 1 g). - The antenna experimental measurements show a 93.3% match between the simulated and measured results.
Kaur and Kaur [92] (2020, Thapar Institute of Engineering and Technology, Patiala, India)	Active, radar-based	Phantom study 3D	3.71–11.48 GHz	Fork-shaped microstrip patch antenna designed using Computer Simulation Tool: Microwave Studio software (CST MWS) V’18.	- The simulated results show that more reflections, lesser specific absorption rate and more conduction current. - Density was obtained in the presence of tumor as compared to a nonmalignant case.
Xiao et al. [93] (2020, Tianjin University, Tianjin, China)	MWI with simulated annealing	Numerical simulation and phantom study 2D, 3D	6 GHz	Patch antenna array working in multi-static mode, pulse pattern generator (Gaussian monocycle pulse), switching matrix, and oscilloscope.	- Owing to simulated annealing algorithm, the proposed method was able to quickly and accurately find the optimal permittivity and achieve the accurate reconstruction of microwave breast image, making the detection process more efficient.
Mehranpour et al. [94] (2020, Imam Khomeini International University, Qazvin, Iran)	Active, radar-based	Phantom study 2D	1.3–6.8 GHz	MARIA system with multi-static hemispherical array of modified UWB bowtie antenna.	- The system successfully reconstructed tumor images (with a small radius of 7 mm). - The proposed high-accuracy calibration (HAC) algorithm was better at detecting the cancerous tumor than the WA and WF methods.
Bocquet et al. [95] (1990, Lille University of Science and Technology)	Passive, radiometry	Clinical trials on 97 patients: normal and malignant tissues 2D	2.5–3.5 GHz	Multi-probe radiometer.	- The acquisition method and software were improved after preliminary experiments on 72 random patients. - For 18 patients, the technique gave good results: the malignant lesions had a radiometric ratio greater than 65%, while the benign lesions were characterized by a ratio smaller than 55%. - Further investigation on seven other patients did not give the same good correlation between the radiometric ratio and the histological characteristics of the tumor.
Carr et al. [35] (2000, East Virginia Medical School)	Passive, radiometry	Clinical trials on 138 patients: malignant tissues -	-	ONCOSCAN system	- Out of the 138 scans, 16 were excluded for technical malfunctions. - There were 40 benign biopsies with positive ONCOSCAN scores. - The positive predictive value of ONCOSCAN was 41% which was higher than that of the mammography (24%).

**Table 4:** Summary of relevant clinical microwave imaging studies for breast cancer detection.

Author (Year, Location)	MWI Method	Study Type & Dimensions	Frequency Range	Measurement System	Findings
Meaney et al. [16,29,37,38,39] (2000–2014, Dartmouth College)	Active, MWT	Phantom study and clinical trials on 500+ patients: normal and malignant tissues 2D, 3D	300 MHz–3 GHz	Monopole antenna array: latest system employed 16 transmitting antennas (Tx) and 15 receiving antennas (Rx) with patient lying in prone position and breast pendant in coupling solution.	- The average relative permittivity of the breast may correlate with radiological breast density labels. - The best results were reported at a frequency of 1300 MHz.- In phantom studies, the reconstructed images of the breast phantoms with tumor-like inclusions were quite discernible.- Clinical trials demonstrated that small tumors could be detected, which confirmed that MWI has potential for early-stage breast cancer detection.- In monitoring the progress of neoadjuvant, changes in microwave properties were noticed which agreed well with the overall NCT treatment response.
Fear et al. [49,50,96] (2003–2012, University of Calgary)	Active, TSAR	Phantom study and clinical trails on 8 patients: normal and malignant tissues 2D, 3D	0.05–15 GHz	A single antenna first scans the pendant breast to determine breast volume compared to tank then a second coronal scan is performed for the tumor detection algorithm. Deconvolution is used to determine the thickness of the skin layer.	- Phantom simulated data showed success in reducing the error percentage in both breast skin location and thickness estimates by more than half.- Clinical results showed that TSAR has an ability to detect and localize tumors with sizes > 4 mm in diameter.
Preece et al. [46,47,48,97] (2008–2016, University of Bristol)	Active, MSA	Phantom study and clinical trials on 86 patients: normal and malignant tissues 2D, 3D	4–10 GHz	16 stacked patch antennas located on a section of a hemisphere to better conform to the curvature of the breast. The patient rested in prone position with breast pendant in a ceramic cup filled with coupling liquid.	- In phantom studies the system was successful at detecting tumors 4 to 6 mm in diameter.- The outcome of the clinical trial with the 31 element prototype was mixed.- The clinical trials with the 60 element system showed improvement in terms of reproducibility and accuracy.
Porter et al. [98] (2016, McGill University, Canada)	Active	Clinical trials on 3 patients	2–4 GHz	Multistatic radar with the 16 sensors embedded in a wearable bra.	- Scans were found to be repeatable, yet many sources of variability were identified, such as patient positioning.
Song et al. [99] (2017, Hiroshima University Hospital, Japan)	Active	Clinical trials on 5 patients 3D	3.1–10.6 GHz	4 x 4 cross-shaped dome antennas array designed to be placed on the breast of a supine. Patient with the breast in contact with a plastic dome covering the antennas.	- The 3D tumor localization in the imaging results are in agreement with the results of histopathology analysis. - The final confocal imaging results were consistent with those of MRI.
Yang et al. [100] (2017, Southern University of Science and Technology, China)	Active	Phantom studies and clinical trials on 11 patients 2D	4–8.5 GHz	Multi-static virtual array with two ultra-wideband horn antennas controlled by mechanical rotation.	- System was sensitive to the increase in the amount of tissue due to cell proliferation.
Kuwahara et al. [101] (2017, Shizuoka University, Japan)	Active hybrid MIST-UWB device	Numerical simulation and clinical trials on 2 patients 3D	1–3 GHz	Breast pendant through an opening in the table directly in contact with stacked patch antennas or a coupling shell of a biocompatible material.	- Data correlation between the measured and calculated data is larger than 0.99. - Images were successfully reconstructed under the experimental conditions.
Rana et al. [102] (2019, London South Bank University)	Active, radar-based	Numerical simulation study and clinical trials: normal and malignant tissues 2D	1–9 GHz	Non-ionizing microwave signals are transmitted through breast tissue and scattering parameters are received via moving transmitting and receiving antenna setup.	- Study differentiated between normal breasts and without lesions breasts. - Results obtained from multilayer perceptron algorithm yielded higher overall specificity compared to results obtained from nearest neighbor algorithm. - The employment of machine learning on clinical data helped the radiologists in the diagnosis process and improved the detection sensitivity.
Sani et al. [103] (2019, Spin off of University of Perugia)	Active	Numerical simulation study and clinical trials: normal and malignant tissues 2D	1–9 GHz	Apparatus constituted by one transmitting antenna and by one receiving antenna.	- The proposed microwave imaging apparatus based on the Huygens principle is safe as it does not require breast compression and does not emit any ionizing radiation.
Song et al. [104] (2020, Hiroshima University)	Active, radar-based	Phantom study and clinical trial on 1 patient: malignant tissues 3D	3.5–15 GHz	Detector composed of a step-motor, a control module, a radio-frequency (RF) module, and a 16-element dome antenna array.	- The proposed method was effective in clutter suppression and improved image quality. - In the clinical test the estimated position of the tumor using the developed system was in good agreement with the physical tumor location examined by MRI and DbPET.
Vispa et al. [105] (2020, University of Perugia, Perugia, Italy)	Active, radar-based	Phantom study and clinical trials on 51 breasts: normal and malignant tissue (7 carcinoma, 9 fibroadenoma, and 5 microcalcifications) 2D	1–9 GHz	Cup to hold breast, horn Tx antenna and microstrip monopole Rx antenna located inside a hub. Tx and Rx antennas connected to a vector network analyzer (VNA).	- Clinical trials showed that microwave images of non-healthy breasts had a mean MAX/AVG of approximately 7% greater than those of the healthy breasts.
Norouzzadeh et al. [106] (2020, K. N. Toosi University of Technology, Tehran, Iran)	Active, transmission radar-based system	Numerical simulation study, and clinical trials on 2 patients: normal and malignant tissue 2D	1–9 GHz	Two low-loss plexiglass plates for breast compression, two UWB bowtie antennas for transmitting and receiving connected to an HP 8720C vector network analyzer. The whole system was controlled by an iPC25 using a Matlab interface.	- For both patients, comparing the microwave image with the X-ray image confirmed tumor existence. - The attenuation of cancerous region was not constant, indicating that cancerous regions have inhomogeneous dielectric properties.

## 4. Image Reconstruction and Quality

### 4.1. Image Reconstruction

Recent advances in computational methods and microwave hardware design are the reasons why many researchers nowadays are considering MWI as a possible cost-effective breast cancer screening modality. One of the important aspects of MWI is the ability to reconstruct images using the collected data, as well as assess their quality.

As mentioned in the previous sections, the objective of MWT is to map the dielectric properties of an OI by collecting measurements at antennas positioned outside the imaging domain [18,107]. Furthermore, as mentioned earlier, the reconstruction of dielectric properties involves solving a mathematical problem that is usually classified as an inverse scattering problem, which are nonlinear, thus requiring iterative methods in order to reach a solution. These equations can be linearized using different approximations, such as the Born approximation. However, in medical imaging the inherent heterogeneity of tissues and high dielectric contrast can lead to non-real solutions. Therefore, the use of iterative optimization algorithms such as the distorted BIM (DBIM), Gauss–Newton inversion (GNI) [108], and the contrast source inversion (CSI) [109,110] methods was proposed. These methods are used to solve the numerical problem, formulated either using integral equations (IEs) or partial differential equations (PDEs). The IE form could be solved using the method-of-moments, whereas the PDE formulation could be solved using the finite-difference method (FDM) or the finite element method (FEM). Each algorithm and formulation has its pros and cons [57,111]. Nevertheless, solving these inverse scattering problems is computationally expensive.

Unlike MWT, UWB radar-based imaging is less computationally demanding because it does not require solving an inverse scattering problem. The quality of a radar-based microwave breast image is largely dependent on the clutter rejection capability of the imaging algorithm and the efficacy of early-time artifact removal methods. Some of the algorithms used to reconstruct images from UWB MWI systems include [112]:

- Delay-and-sum (DAS);
- Delay-multiply-and-sum (DMAS);
- Improved delay-and-sum (IDAS);
- Coherence-factor-based DAS (CF-DAS);
- Channel-ranked DAS (CR-DAS);
- Robust Capon beam former (RCB).

Sagheer et al. [113] compared the image reconstruction performance of DAS and DMAS. Although DAS is a simple MWI reconstruction algorithm with real-time execution, the experimental results illustrated that it had limited capabilities in suppressing noise and the artifacts. On the other hand, DMAS offered high-contrast resolution but required longer computational time. The authors in [112] conducted a study in which they inspected the performance of the aforementioned reconstruction algorithms in the presence of noise by using experimental breast phantoms. The breast phantoms employed in this study were developed at the University of Calgary and scanned using a TSAR system prototype. The image quality metrics used to evaluate these algorithms were the signal-to-clutter ratio (SCR), the signal-to-mean ratio (SMR), and localization error [112]. SCR is defined as the ratio of tumor intensity to clutter intensity in the image. SMR is defined as the ratio of the average intensity of the tumor region to the overall image. The localization error is the distance between the known tumor location and the detected location. A follow-up to the aforementioned study in [114] was conducted where the six imaging algorithms were used to reconstruct 3D images of five clinical patients. The results showed that DAS was able to detect most malignancies; however, clutter was observed in the obtained images. IDAS and CF-DAS improved the image quality, but they often failed to correctly localize the malignancy, especially when there were multiple lesions and/or heterogeneous breast tissue. CR-DAS did not provide significant improvement compared to DAS. In addition, RCB suffered from the presence of coherent interferences from multiple lesions and the heterogeneity of the breast.

### 4.2. Reconstruction Quality

Image quality can determine the accuracy of the imaging test and influence the clinicians’ diagnostic decision. Recent improvements in imaging techniques and instrumentation have improved early diagnosis and treatment. However, a quantitative method is needed to assess these technological advances.

Quantitative assessments of diagnostic images’ quality are usually reported in terms of spatial resolution, signal-to-noise ratio (SNR), and contrast-to-noise ratio (CNR). Spatial resolution refers to the ability of an imaging system to differentiate between closely spaced objects/features. Spatial resolution is expressed mathematically by line-spread functions (LSFs) and point-spread functions (PSFs). Generally speaking, the narrower the LSF the higher the spatial resolution, and for a perfect representation of the object, the PSF would be a delta function in all three dimensions [115].

Certain operating and design parameters, such as the operating frequency and number of antennas in the array, affect the quality of the reconstructed images. Given the novelty of MWI in biomedical applications, the effect of these parameters on the spatial resolution, SNR, and CNR is not fully understood yet. Several studies have been conducted in an attempt to determine just how different design and operating parameters affect the reconstructed image quality. This topic is considered at the forefront of challenges which, once resolved, would make MWI systems a serious contender in the medical imaging circles. In a study conducted by Chang et al. [116], the spatial resolution of microwave images was found to be dominated by the bandwidth of the microwave signal. According to their study, the higher the chosen frequency, the higher the image quality. However, at high frequencies, the signals propagating through the tissue will experience serious attenuation. As such, the choice of the operation frequency requires a trade-off between resolution and penetration depth. The study showed that the attenuation is about 4 dB/cm when microwaves penetrate the tissue. Regarding SNR, Nikolova and McCombe [69] proposed a method to assess the SNR of MWI acquisition systems. The approach evaluates noise by taking the standard deviation of the signal in the feature-free region (a region where no signal is expected), while the signal strength is the mean value in the region of interest (ROI) in a calibration object whose contrast distribution is already known.

## 5. Challenges and Future Research Directions

As mentioned in earlier sections, MWI is a promising medical imaging modality that can be a cost-effective alternative to existing imaging modalities. However, most of the feasibility studies carried out so far were performed through numerical simulations, which are not enough to consider MWI systems in clinical settings. As seen from the discussion of the research studies relevant to this technology, clinical application to MWI has been attempted by some research groups. However, there are still many technical challenges facing MWI as a breast cancer detection tool that still need to be addressed to improve this technique and achieve its full capacity. Several research directions are discussed in the following subsections [11].

### 5.1. Effective Coupling of Microwave Signal

For tomographic systems, a big challenge lies in effectively coupling the transmitted microwave signal to the body.This can be attributed to the vast differences in dielectric properties between the breast tissue and the medium in which the antennas are placed. As a result, strong reflections occur at the tissue–medium boundary, weakening the signal penetrating the body. When the weak scattered signals are used for imaging, a very large dynamic range is produced which results in having some low-frequency signals attenuated or completely lost. An approach to minimize coupling effects is to use a more impedance-compatible coupling liquid. Meaney et al. [37,38,39] substituted saline with a water–glycerin mixture as a coupling medium. They investigated different ratios of water and glycerin and found that an 83:17 glycerin to water mixture produced nominal coupling effects [38]. A research group at the University of Bristol designed an antenna in a hemispherical shape to better fit the curvature of the breast [46,47,48]. Felício et al. [82] proposed a radar-based MWI method using a dry setup that does not require a coupling liquid. In their method, a webcam was used to estimate the breast three-dimensional surface information and its distance to the antennas. This information was incorporated into a signal processing algorithm based on singular value decomposition to remove the skin backscattering caused by the absence of coupling liquid.

### 5.2. Contrast Agents

MWI is based on the fact that a significant difference in the electrical properties of malignant and healthy tissues exists. However, if this difference is small, detecting tumors using MWI becomes a much more challenging task. A proposed solution to this problem is to use contrasting agents. Contrary to healthy tissues, the leaky vasculature of tumors facilitates the diffusion of contrasting agents into tumors, enhancing their electrical properties. For example, Bucci et al. [41] used MNPs as their contrasting agent. The method is based on the reconstruction of the magnetic contrast induced by the MNPs in the presence and the absence of the polarizing magnetic field. The findings of this study were quite promising, as significant enhancement in contrast was observed. Another example is the recent work carried out by Kaye et al. [117], where MNPs were used to enhance breast imaging inside a ferromagnetic resonance imaging chamber.

### 5.3. Signal Processing and Imaging Algorithms

In MWI, breast tumor detection is performed by measuring the backscattered data which may include skin backscatter and late time clutter response. Thus, robust signal processing is required to remove the unwanted signals. Moreover, quantitative MWI algorithms are known to have inherent challenges. For example, in solving inverse problems, which are needed for MWT, issues such as nonlinearity and the ill-posedness arise. Oscillations can occur in the estimated electrical properties at the boundary of the high-dielectric-contrast tissues, even with the application of different regularization methods. Recently, machine learning and deep learning algorithms have been used to create intelligent decision-support systems to help clinicians recognize malignant lesions in breasts. For instance, convolutional neural networks (CNN or ConvNet) can be fed multiple scattering data as the forward-pass; then the CNN learning algorithm combines the extracted features and aggregates them in a nonlinear fashion to predict the output. The advantages of CNN-based learning include improved computational efficiency, especially when solving inverse scattering problems. Examples of studies that used machine learning and deep learning neural networks to simplify the inverse problem are given in [102,118,119,120,121]. In addition, a 2D-based imaging algorithm may not work for a 3D biological object and might lead to inaccurate reconstruction results. For instance, when cylindrical phantoms are used in 2D studies, ideally the heights of the used cylinders must be infinitely large for the approximations to be applicable. However, this is not feasible in practical applications; thus, phantoms of finite heights will affect the accuracy of the results obtained. This highlights the need for 3D modeling; however, 3D imaging algorithms are usually computationally expensive. Thus, further work is required to develop inversion algorithms with higher efficiency and accuracy [11,122].

### 5.4. Antennas and Measurement System

As mentioned in the previous section, ill-posedness is one of the problems encountered in solving the inverse problem. To address this issue, some researchers proposed increasing the number of antennas used to acquire larger scattered field datasets [11]. However, there is a physical limitation on the number of antennas that can be used. Moreover, placing the antennas too close to each other may result in high mutual coupling between the antennas, introducing errors in the measured scattered field. One of the conventional methods to address antenna coupling involves inserting a decoupling slab between the radiating antennas. Other proposed techniques include the use of cavity-backed substrate removal, defected ground structures, metamaterial insulators, slotted complementary split-ring resonators, defected wall structure, and employing electromagnetic band gap structures between antennas [123]. Other errors that might be introduced in a measurement setup are cable losses, phase shifts, or mismatch at the connectors [124]. A calibration process to remove some of these errors was suggested by Gilmore et al. [124]. The authors also provided a method to avoid the frequencies where the coupling is large enough to prevent successful imaging in a wideband measurement setup. Another method to avoid coupling is to use a virtual array to scan the body, but this comes at the cost of increased scan time. Therefore, the design of a measurement setup having an optimum scan time, low error, and antennas having a minimal mutual coupling, as well as effective calibration methods for the system, can be a future research direction [11].

### 5.5. Frequency Bandwidth and Resolution

Another important issue in MWI is choosing a suitable imaging frequency. As discussed in Section 2, MWI is used in many different applications, each of which requires a different operating frequency. In MWI, the choice of a suitable frequency bandwidth is a trade-off between penetration depth and resolution. The depth of penetration is inversely proportional to the frequency due to the increased attenuation in the tissues at relatively high frequencies. However, the resolution is proportional to the frequency because of the decrease in wavelength and pulse durations accompanying increased operating frequencies. Thus, different frequencies are used to image different body parts due to the difference in thickness of the body part being imaged and the tissue compositions. Hence, further investigations are required to achieve a standardized optimum frequency and an acceptable resolution [11].

### 5.6. Interference and Noise

Clinical environments are ones in which there are significant amounts of electromagnetic interference. Using an MWI system in a clinical environment is a challenging task as it will be affected by the electromagnetic interference and noise from sources such as mobile phones, wireless local area network, other radiology equipment, fluorescent lights, computers, and noisy electrical power supplies. Surges may also contribute to interference and noise in a medical facility [125]. Thus, further research is required to develop MWI systems that are robust against interferences as well as electromagnetically compatible with other medical systems [11,125].

### 5.7. Hybrid and Portable Systems

Some researchers are investigating the coupling of MWI with ultrasound transducers in hybrid microwave–acoustic imaging [126,127]. Another direction that is being explored is compressive sensing to improve the specificity, sensitivity, and accuracy of breast cancer diagnosis [11]. Moreover, developing portable MWI devices is an area that has seen a lot of interest recently, given the benefits of portable imaging devices for patients’ comfort and medical preparedness in cases of natural disasters. Researchers at Hiroshima University investigated the possibility of developing a handheld impulse-radar breast cancer detector and reported promising results [99]. Adachi et al. [128] reported on a radar-based portable MWI system for detecting breast cancer. The results showed that the system can be safely used and that it showed great potential in detecting breast cancer, including non-invasive ductal carcinoma and micro-invasive carcinoma, which were not as easily detected by mammography due to dense breast tissue. Other reports on portable MWI systems are detailed in [76,129,130,131,132].

### 5.8. Millimeter Wave Imaging

Recently, there has been increased interest in millimeter wave (mm-wave) frequencies, as this represents a natural continuation of the research path at microwave frequencies. In addition, this is facilitated by the technological advances in the design of transmission and reception systems that function at those mm-wave frequencies, as well as cost reduction. One of the pioneers in this field is the research group at the University of Pavia that investigated the use of mm-wave for breast cancer imaging [133]. The system was operated at a frequency of 30 GHz and it was demonstrated that a penetration depth of a few centimeters was achieved. Moreover, the authors showed that a conformal system layout is better than the linear layout, as the target position was identified in the breast model at a depth of around 41 mm, which can be considered in good agreement with the theoretical depth of 40 mm. In another study, Iliopoulos et al. [87] proposed a field-focusing technique based on a convex optimization method to improve the penetration of mm-waves into tissue-mimicking phantoms. The theoretical and simulation results were in good agreement, which validated the overall approach.

## 6. Conclusions

In conclusion, breast cancer is a serious threat to women’s health worldwide. Numerous research studies have stressed the significance of early detection in the management and treatment of breast cancer. Although current imaging methods used in breast cancer screening are effective, they still have some limitations. MWI, as an early breast cancer detection tool, has attracted increased interest from researchers worldwide. With a variety of promising approaches (passive radiometry and active, which in turn includes tomography and radar-based imaging) and several research groups involved, there are good reasons to believe that microwave breast cancer detection will become a successful screening tool either as a standalone technology or as a complement to conventional mammography. This paper provided an overview of the rationale, methods, advances, and ongoing research of MWI in breast cancer detection applications. An exhaustive discussion of the working principle behind MWI along with both its passive and active variations was provided. Moreover, a classification of some of the most pertinent studies to this technology was presented. The paper showed that the reviewed numerical, experimental, and clinical studies reported promising results. Nonetheless, there is still an enormous amount of research and development to be carried out in order to achieve the full capacity of this technology. The paper discussed the challenges and future research directions that still need to be addressed to improve MWI in clinical applications, including better hardware design, signal-processing methods, and image reconstruction algorithms.

## Figures and Tables

**Table 1:** Dielectric properties of female breast tissue at 3.2 GHz (adapted from Campbell and Land [24]).

Tissue Type	Relative Permittivity	Conductivity (mS/cm)	Water Content (%)
Fatty tissue	2.8–7.6	0.5–2.9	11–31
Normal tissue	9.8–46	3.7–34	41–76
Benign tissue	15–67	7–49	62–84
Malignant tissue	9–59	2–34	66–79

**Table 2:** Chronological summary of relevant studies for dielectric properties of breast tissues.

Study (Year)	Measurement Technique (Temperature °C)	Number of Samples	Frequency Range	Tissue Type	Findings
Chaudhary et al. [22] (1984)	RX-meter and TDS probe: Samples collected in physiological saline and are in inserted into RX-meter chamber or pressed against using TDS probe (25 °C).	15	3 MHz–3 GHz	healthy and malignant tissues.	- Malignant tissue displayed 3–5 times increase in electrical properties compared to healthy tissue. - The greatest permittivity difference occurred at frequencies less than 100 MHz.
Surowiec et al. [23] (1988)	Open-ended coaxial capacitive sensor: Samples inserted into a holder (37 °C).	7	20 kHz–100 MHz	healthy and malignant tissues.	- Central region of tumor and surrounding tissue yielded higher dielectric values than peripheral tissue.
Campbell and Land [24] (1992)	Resonant cavity perturbation method: Samples inserted into holder (unspecified).	37	3.2 GHz	healthy, malignant, and benign tissues.	- Cancerous tissue showed higher dielectric properties. - Difference in dielectric properties between benign and malignant tumors not sufficient to differentiate between them.
Joines et al. [25] (1994)	Open-ended coaxial probe: Samples pressed against using probe (24 °C).	12	50–900 MHz	healthy and malignant tissues.	- An average difference in permittivity and conductivity of 233% and 577%, respectively, was observed between healthy and cancerous tissues.
Meaney et al. [29] (2000)	Radiating monopole antenna array submerged in saline bath surrounding the breast (25 °C).	5	900 MHz	healthy tissue.	- Average permittivity values at 900 MHz were significantly higher than those reported by Joines et al. [25]. - A correlation was established between fat content and the average permittivity values.
Choi et al. [26] (2004)	Open-ended coaxial probe: Samples pressed against using probe (unspecified).	12	0.5–30 GHz	healthy and malignant tissues extracted from lymph nodes.	- Significant contract in dielectric properties between healthy and malignant lymph nodes’ tissues.
Lazebnik et al. [27,28] (2007)	Open-ended coaxial probe: Samples pressed against using probe. (18–25.70 °C in University of Wisconsin) (19.5–26.60 °C in University of Calgary).	354, from 93 patients [27] and 196 patients [28]	0.5–20 GHz	healthy and malignant tissues extracted from adipose, fibro connective and glandular regions of the breast.	- Dielectric properties of breast tissues are primarily determined by the adipose content. - The location from which the healthy tissue sample is taken affects the comparison. - Significant contrast, 10:1, in dielectric properties between healthy and malignant tissues.
Martellosio et al. [30] (2017)	Reflectometry. Open-ended coaxial probe and VNA (25 °C).	220	0.5–50 GHz	healthy and malignant tissue	- Dielectric properties of normal tissues showed wider variability than the tumorous tissues. - Performance comparable to that of mammography performed in vivo on patients. - Tumorous tissues had higher real and imaginary parts of the complex permittivity than normal breast tissues.
Meo et al. [31] (2017)	Reflectometry. Open-ended coaxial probe and VNA (25 °C).	124	0.5–50 GHz	healthy and malignant tissue	- The results for sensitivity were 90% both for real and imaginary part, while those for specificity were 75%.
Meo et al. [32] (2018)	Reflectometry. Open-ended coaxial probe and VNA (−40–220 °C).	346	0.5–50 GHz	healthy and malignant tissue	- Higher variability in dielectric properties of healthy tissues compared to malignant ones.

## Data Availability

Not applicable.

## Abbreviations

The following abbreviations are used in this manuscript:

BIM	Born Iterative Method
CF DAS	Coherence Factor Based Delay-And-Sum
CMI	Confocal Microwave Imaging
CNR	Contrast-to-Noise ratio
CR DAS	Channel Ranked Delay-And-Sum
CS	Compressive Sensing
CSAR	Circular Synthetic Aperture Radar
DAS	Delay-And-Sum
DBIM	Distorted Born Iterative Method
DMAS	Delay-Multiply-And-Sum
FDG	Fluorodeoxyglucose
FDTD	Finite Difference Time Domain
FEM	Finite Element Method
FIR	Finite Impulse Response
HMI	Holographic Microwave Imaging
HMIA	Holographic Microwave Imaging Array
IDAS	Improved Delay-And-Sum
IC-DAS	Iteratively Corrected Delay-And-Sum
kNN	k-Nearest Neighbor
LSF	Line Spread Function
MIST	Microwave Imaging via Space Time
MITAI	Microwave-induced thermoacoustic imaging
MLP	Multilayer Perceptron
MNP	Magnetic Nanoparticles
MRI	Magnetic Resonance Imaging
MSA	Multistatic Adaptive
MWI	Microwave Imaging
MWT	Microwave Tomography
NCT	Neoadjuvant Chemotherapy
PDE	Partial Differential Equation
PET	Positron Emission Tomography
PSF	Point Spread Function
RCB	Robust Capon Beamformer
ROI	Region of Interest
SAR	Synthetic Aperture Radar
SCR	Signal-to-Clutter Ratio
SMR	Signal-to-Mean Ratio
SNR	Signal-to-Noise Ratio
SVM	Support Vector Machine
TDS	Time Domain Spectroscopy
TSAR	Tissue Sensing Adaptive Radar
US	Ultrasound
UWB	Ultra Wideband
WHO	World Health Organization
